# Detection of short repeated genomic sequences on metaphase chromosomes using padlock probes and target primed rolling circle DNA synthesis

**DOI:** 10.1186/1471-2199-8-103

**Published:** 2007-11-13

**Authors:** Jakob S Lohmann, Magnus Stougaard, Jørn Koch

**Affiliations:** 1 Institute of Pathology, Aarhus University, 8000 Aarhus C, Denmark

## Abstract

**Background:**

In situ detection of short sequence elements in genomic DNA requires short probes with high molecular resolution and powerful specific signal amplification. Padlock probes can differentiate single base variations. Ligated padlock probes can be amplified in situ by rolling circle DNA synthesis and detected by fluorescence microscopy, thus enhancing PRINS type reactions, where localized DNA synthesis reports on the position of hybridization targets, to potentially reveal the binding of single oligonucleotide-size probe molecules. Such a system has been presented for the detection of mitochondrial DNA in fixed cells, whereas attempts to apply rolling circle detection to metaphase chromosomes have previously failed, according to the literature.

**Methods:**

Synchronized cultured cells were fixed with methanol/acetic acid to prepare chromosome spreads in teflon-coated diagnostic well-slides. Apart from the slide format and the chromosome spreading everything was done essentially according to standard protocols. Hybridization targets were detected in situ with padlock probes, which were ligated and amplified using target primed rolling circle DNA synthesis, and detected by fluorescence labeling.

**Results:**

An optimized protocol for the spreading of condensed metaphase chromosomes in teflon-coated diagnostic well-slides was developed. Applying this protocol we generated specimens for target primed rolling circle DNA synthesis of padlock probes recognizing a 40 nucleotide sequence in the male specific repetitive satellite I sequence (DYZ1) on the Y-chromosome and a 32 nucleotide sequence in the repetitive kringle IV domain in the *apolipoprotein(a) *gene positioned on the long arm of chromosome 6. These targets were detected with good efficiency, but the efficiency on other target sites was unsatisfactory.

**Conclusion:**

Our aim was to test the applicability of the method used on mitochondrial DNA to the analysis of nuclear genomes, in particular as represented by metaphase spreads. An optimized protocol for chromosome spreading in diagnostic well-slides was used for the detection of circularized padlock probes amplified by target primed rolling circle DNA synthesis from condensed metaphase chromosomes. We were able to detect a 40 nucleotide sequence in the male specific repetitive satellite I sequence and a 32 nucleotide sequence in the repetitive kringle IV domain in the *apolipoprotein(a) *gene. Our overall conclusion is that whilst this type of reaction indeed can be brought to work on nuclear genomes, including metaphase chromosomes, the total efficiency of this multistep reaction is at present relatively low (1–10% of target sites picked up), meaning that it is best suited for the detection of targets that exist in multiple copies per cell. Changing this will require substantial efforts to systematically increase the efficiency in each step.

## Background

Molecular dissection of in situ hybridization targets whereby short regions of larger elements are investigated at high molecular resolution is of obvious value in research and diagnostics. A design for this purpose was provided by Koch et al. [1], in the form of the PRINS technique. This technique employs oligonucleotide probes, which hybridize to complementary sequences in the specific target, and a DNA polymerase to achieve DNA synthesis specifically at sites of probe binding (see also [2,3]). Such labeling through synthesis eliminates the direct relationship between probe size and signal intensity, allowing the more widespread use of oligonucleotide probes, which due to their small size may resolve small sequence variations in potential target sequences. As a result of the signal enhancement of the PRINS design it is thus possible to analyze sequence structures within primate alpha satellite DNA [1] and demonstrate single base variations in such DNA [4]. A limitation to the original design was that the extent of the chain elongation (a few hundred basses) was insufficient for the detection of single probe molecules. Thus, whilst the reaction worked exceedingly well for the detection of tandem repeated sequences, where multiple probes/primers would hybridize within the same area to provide a summation of signals, results on single copy targets have generally been disappointing. In addition to the size of the extension product, the sensitivity problem also relates to the issue of background staining – unavoidable breaks in the genomic DNA will produce 3'-ends capable of initiating a nick translation in situ, where the extension product will be similar to that originating from a hybridized probe/primer. Attempts to reduce this background through pre-ligation of the slide or pre-extension with dideoxy-nucleotides were only successful on older, more damaged preparations, but inclusion of a dideoxy-nucleotide in the PRINS reaction mixture reduced it by an order of magnitude. Whilst this would also block most PRINS reactions, some targets, such as telomeric repeats or trinucleotide repeats, in fact miss one or more of the four DNA bases. Including the missing base(s) as a dideoxy-analogue provided an extremely sensitive detection reaction – though with limited application [5,6].

We here report another approach, in which linear oligonucleotide probes are replaced by circular probes – so-called padlock-probes [7] – and the signal generating DNA synthesis reaction is performed in a rolling circle format giving improved specificity and enhancement. A padlock probe is a single stranded oligonucleotide of approximately 70 nucleotides, which upon hybridization to a target sequence has its ends brought into proximity [7]. The two ends can be ligated, thereby turning the probe into a single stranded circle, which is locked to the substrate. Furthermore, if a high salt concentration is maintained during the ligation step, T4 DNA ligase can differentiate base pair variations at the point of ligation with very high precision [8,9].

The closed circular nature of ligated padlock probes makes it possible to use them as templates for rolling circle DNA synthesis; rolling circle DNA synthesis of artificial small circular DNAs was reported more than a decade ago by Fire and Xu [10]. Coupling rolling circle DNA synthesis to the PRINS design equips the latter with the high productivity of the rolling circle format and should provide enough localized DNA synthesis for the detection of individual probe molecules in a format (rolling circle DNA synthesis) that can be discriminated from endogenous DNA synthesis.

**Figure 1:**
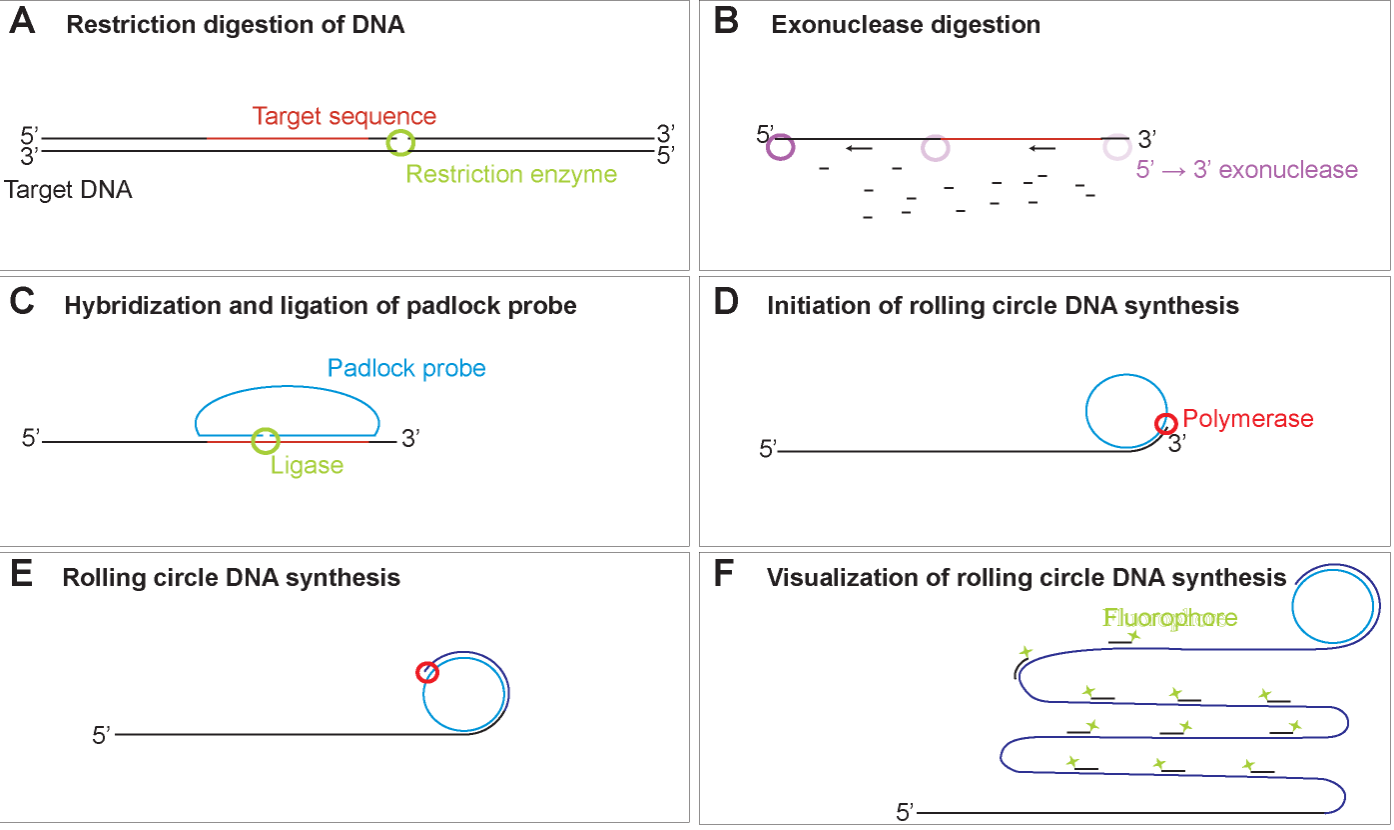
**In situ detection of DNA using padlock probes and target primed rolling circle DNA synthesis**. **(A) **The samples are cleaved with a restriction enzyme having a restriction site positioned 3' to the probe binding sequence. It is important that the enzyme does not have any other cleavage sites in close proximity to the 5'-end of the probe binding sequence to avoid degradation of the recognition sequence during exonuclease treatment. **(B) **The target sequence is made single stranded using the lambda exonuclease which digests duplex DNA in the 5'→3' direction in a highly processive manner, thereby making the target sequence single stranded. **(C) **The padlock probe is hybridized and ligated on the target sequence. Only padlock probes which are correctly hybridized at the point of ligation will be circularized. **(D-E) **The rolling circle reaction is initiated by using the target sequence as a primer, thereby locking the rolling circle product to the target sequence. **(F) **The rolling circle product is visualized by hybridizing a labeled oligonucleotide to the part of the padlock probe not recognizing the genomic hybridization target.

However, to achieve this combination it is necessary to overcome several potential problems. Firstly, linking of the ligated padlock probe to the target, may make it unlikely to roll efficiently in an in situ format [11]. Secondly, amplification by rolling circle DNA synthesis has the disadvantage that there may be a spreading of the signal away from the point of hybridization, if an external primer is used. Thus, spatial resolution is lost, similar to the situation that is observed in in situ PCR [12]. A solution to these problems were first presented in a patent application by J. Koch [13] and subsequently elaborated on in collaboration with the group behind the padlocks probes within the MolTools consortium under EU-framework 6 [14]. This method involves cleaving the target DNA 3' to the padlock probe to provide a free 3'-end for the initiation of DNA synthesis, to unlock the padlock probe so it may roll freely, and to covalently link the rolling circle product at the site of synthesis. The details of the design were published by Larsson et al. who presented a method for the detection of single nucleotide differentiation in mitochondrial DNA in situ using target primed rolling circle DNA synthesis (Figure 1) [9]. However, since each cell contains hundreds of mitochondria, with each mitochondrion containing numerous genomes, and since mitochondrial DNA is not as tightly packed as genomic DNA, it remained an open question whether the approach could also be applied to less numerous targets. Furthermore, it is not necessarily straightforward to adapt the method from mitochondrial detection to in situ analysis of nuclear genomes. Indeed, previous attempts to amplify padlock probes from condensed metaphase chromosomes have been unsuccessful [15], and the group presenting this study later published a follow-up study in which staining in interphase nuclei was interpreted as a positive signal, but no signals appeared on metaphase chromosomes [16].

We present here target primed rolling circle DNA synthesis of padlock probes on condensed metaphase chromosomes spread in teflon-coated diagnostic well-slides. We were able to detect the male specific part of the repetitive human satellite I sequence (DYZ1) positioned on the Y-chromosome and the repetitive kringle IV domain from the *apolipoprotein(a) *gene (*LPA*) positioned on the long arm of chromosome 6 [17,18] with good efficiency.

## Results and discussion

### Chromosome spreading in well slides

We have previously observed that enzymatic DNA amplification reactions in situ may consume sufficient reagents for local depletion to occur, limiting the reaction if it is performed in a standard format in which the liquid phase is a thin film spread under a coverslip [19]. Therefore, after initial experimentation with the standard system, we converted to a well-based system, since it offers several advantages: 1) A larger volume of reaction mixture can be applied per mm^2 ^of sample, to prevent local depletion of reagents. 2) Less reagent per reaction is necessary compared to standard slides. 3) It is easier to multiplex the reactions.

**Figure 2:**
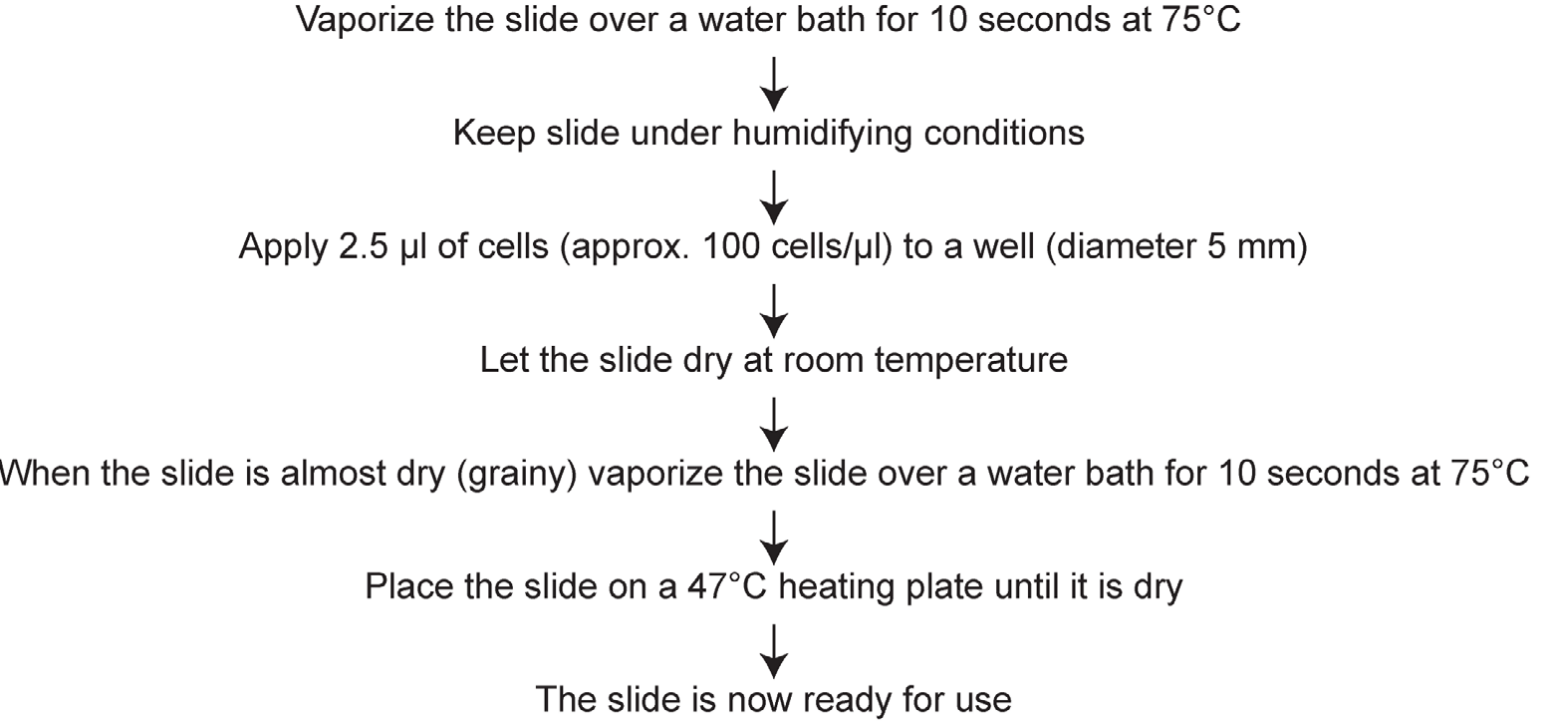
Protocol for spreading metaphase chromosomes in teflon-printed diagnostic well-slides.

**Figure 3:**
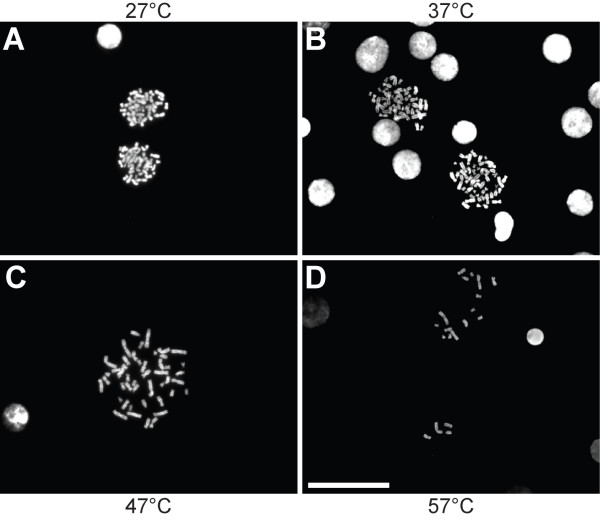
**Metaphase chromosomes spread in diagnostic well slides**. Following the second incubation over hot water vapor, the slides were incubated at different temperatures on a heating plate. **(A) **27°C, **(B) **37°C, **(C) **47°C and **(D) **57°C. Scale bar, 100 μm.

Initially, we encountered problems due to poor spreading of the condensed chromosomes in well-slides. Subsequently, we designed a protocol optimized to well-slides, based on observations by Henegariu et al. [20]. We found that a combination of hot water vapor, low volume of cell suspension, and heating gave the best results (Figure 2), although the result varied from spread to spread. In particular, the temperature following the second incubation over hot water vapor was important to achieve good spreading of the chromosomes. When the chromosomes were dried at 27–37°C they did not spread enough to make a clear distinction between each individual chromosome, whereas too high a temperature (above 57°C) resulted in over-spreading of the chromosomes. The optimal temperature was in general 47°C (Figure 3).

### In situ detection of genomic sequences

We wanted to test whether the in situ padlock system presented by Larsson et al. for the analysis of mitochondrial DNA in situ [9] (see also Figure 1) could be used on condensed metaphase chromosomes. A limitation to this technique is that it involves many enzymatic steps. If each step has for example an efficiency of only 50% it will give a final detection level of 0.5^X^, where × is the number of enzymatic steps. Thus, with four enzymatic steps the success rate will only be 6%, a figure for the overall efficiency that matches well with the 1–10% range estimated in the Larson study. Indeed, in accordance with this estimate, we had disappointing success rates with single-copy genes (data not shown). This indicates that this method is, at present, best suited for targets represented in several copies in the sample, such as mitochondria, chloroplasts, viruses, and, as presented here, repeated sequences in the genome.

The method worked well with two repeated genomic targets, first of which was the Y-chromosome specific part of the satellite I repeat which contains a 2.7 kb dispersed repeat of high copy number (approximately 2000 copies) [17,21]. While this target is a repeated sequence, the hybridization target consists of 2000 single copy sequences spread over the long arm of the Y-chromosome. Thus, with an overall reaction efficiency of 1–10%, 20–200 probes/chromosome should lead to the formation of visible rolling circle products. These should appear as discrete single products or larger conglomerates of products where multiple reactions took place close enough to each other for the signals not to be discriminated in the microscope. Furthermore, the expected number of signals per chromosome would seem high enough that all Y-chromosomes should appear labeled, despite unavoidable random variation across the preparation. The second target we detected with high efficiency was the kringle IV domain from the *LPA *gene sequence. The *LPA *gene encodes the major component of the plasma protein complex lipoprotein(a) (LP(a)) and is in itself a single copy gene. However, it contains a 5.5 kb kringle IV domain which is a repetitive sequence varying in copy number between alleles (12–51 copies per allele) [22]. Thus, while the target is a single copy gene, the hybridization target is contained within a tandem repeat found in 12–51 copies, which with an overall reaction efficiency of 1–10% should give rise to something like 1–5 rolling circle products per chromosome. With the unavoidable random variation across the preparation, some chromosomes (or chromatids) might not harbor a signal, but overall signals should be found at most sites. The copy-number of the kringle IV domains is inversely correlated to the concentration of plasma LP(a) and the risk for coronary heart disease (CHD) [23], so a versatile means of getting a direct measure of the copy number in a particular individual could potentially be of practical interest, and PRINS has previously proven useful for the sizing of telomeric and trinucleotide repeats in situ on individual chromosomes [5,6].

**Figure 4:**
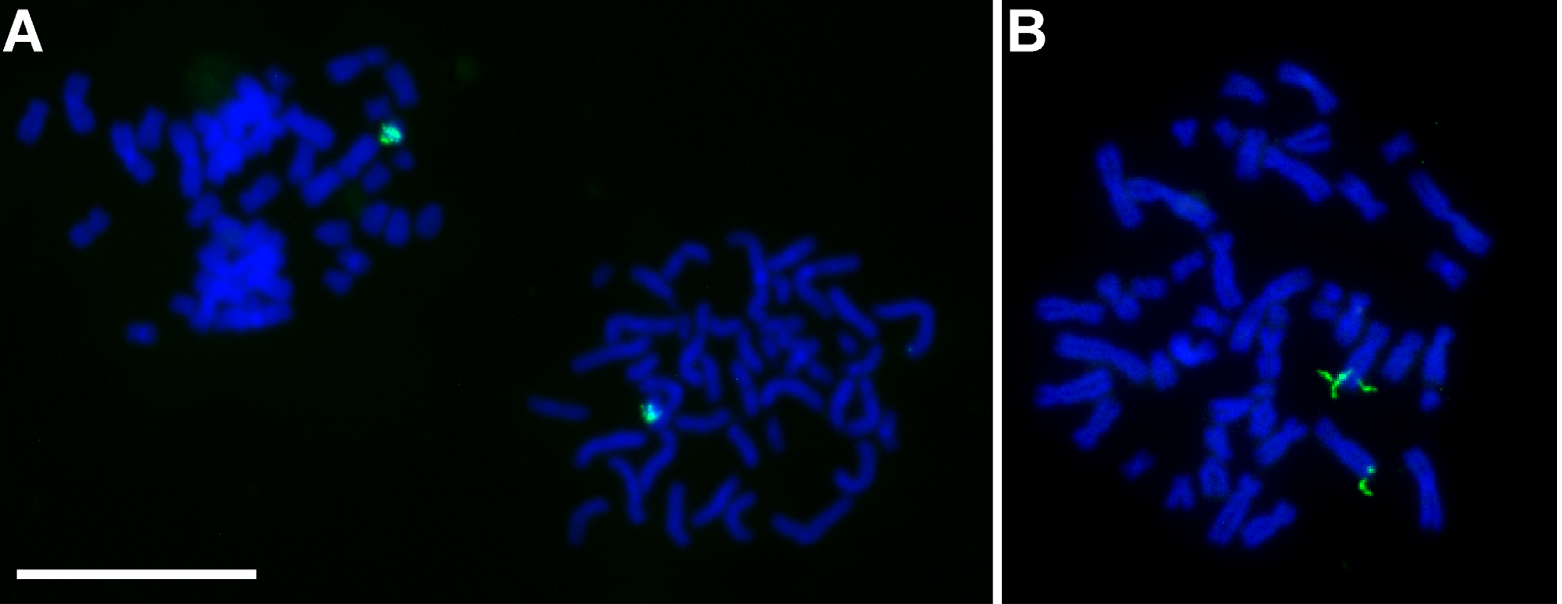
**Detection of a 40 nucleotide sequence in the male specific satellite I repeat and a 32 nucleotide sequence in the *LPA *gene**. Padlock probes were hybridized and ligated to pre-treated chromosomes, followed by target primed rolling circle DNA synthesis. **(A) **Site specific detection of a 40 nucleotide sequence in the male specific satellite I region on the long arm of the Y-chromosome. **(B) **Site specific detection of a 32 nucleotide sequence of the kringle IV domain in the *LPA *gene. It seems that individual rolling circle reactions can produce products long enough to be resolved as threads of DNA in the microscope. Scale bar, 100 μm.

The target sequences for satellite I repeat and for the *LPA *gene were extracted from previously published articles [17,24]. Representative results with padlock probes targeting short sequence elements in the male specific satellite I repeat and in the kringle IV domain in the *LPA *gene are shown in Figure 4.

Using the Y-chrom probe, which has a 40 nucleotide target sequence, the long arm of the Y-chromosome was clearly labeled. All Y-chromosomes were labeled and the signal on any one Y-chromosome appeared as a grainy coloring, each grain representing a rolling circle product, or a set of rolling circle products, covering almost the entire long arm of the chromosome, consistent with the known distribution of the satellite I repeat (Figure 4A). No signals were detected from any other chromosomes, indicating that the correct recognition sequence was selectively detected. The exact number of signals per chromosome was difficult to establish, since the rolling circle products were quite numerous, overlapping, and of variable intensity, but the overall results seemed well in agreement with the 1–10% efficiency observed on mitochondrial DNA.

The signals from the probe for a 32 nucleotide sequence in the kringle IV domain of the *LPA *gene were positioned on the appropriate part of the long arm of chromosome 6. Two distinct signals, one on each sister-chromatid, were ideally obtained from each chromosome 6 (Figure 4B), although in a larger screen, sister-chromatids (of chromosome 6) also appeared with 0 and 1 signals (data not shown). In 70 out of a 100 metaphases both long arms on both chromosome 6 were stained, whereas in the remaining 30 metaphases one or – occasionally – two signals were missing. The overall efficiency could not be estimated with any great precision since: 1) The precise number of kringle IV domains positioned on each allele was not known in our study material. 2) In some chromosome spreads the sister-chromatids could not be differentiated. 3) Individual rolling circle products on one chromatid could generally not be differentiated from each other, because they were situated too closely on the chromosomes.

In summary, we have demonstrated stable detection of short repeated genomic sequences in metaphase chromosomes using padlock probes and target primed rolling circle DNA synthesis, a possibility that seemed questionable based on the literature, and with an overall reaction efficiency in the same range as that previously reported for mitochondrial DNA in situ. Since the overall efficiency is still low, the method seems at this stage of development best suited for the detection of motifs within multi-copy sequences such as repeated sequences in the genome (as presented here), viral DNA, chloroplasts and, as previously described, mitochondrial DNA [9]. It follows from the math of the reaction that adapting it for single target detection would require systematic work to significantly improve the efficiency in each step of the reaction. This may be quite laborious and time consuming, and though the outcome of such an effort, if successful, may be very valuable, it was quite beyond the resources of this study. However, in a parallel study we have addressed the issue of improving one step in the reaction, the ligation of the padlock probes to closed circles. For this we have developed a procedure enabling us to replace the chemically synthesized padlock probes with enzymatically produced oligonucleotides, which have been shown to posses an increased ligation efficiency [25].

## Conclusion

We demonstrate the detection of short sequence elements in situ on condensed metaphase chromosomes using padlock probes and target primed rolling circle DNA synthesis and provide the protocol. The reactions were shown to work best in teflon-printed diagnostic well-slides, this being a preferred format for the enzymatic reactions. The reaction provided efficient detection of a 40 nucleotide sequence in the repetitive satellite I sequence positioned on the long arm of the Y-chromosome, and a 32 nucleotide sequence in the kringle IV domain in the *LPA *gene positioned on the long arm of chromosome 6, while the efficiency on other target sites was unsatisfactory. The method at this stage of development seems best suited for the detection of motifs within multi-copy sequences in the genome (as presented here), viral DNA, chloroplasts and, as previously described, mitochondrial DNA [9].

## Methods

### Oligonucleotides

All oligonucleotides were purchased from DNA Technology A/S, Aarhus, Denmark and are listed in Table 1.

**Table 1:** Oligonucleotide list

**Name**	**Sequence**
Apo(a)	5'-P-GAGGCACATA CTCCATTTAT TTCCTCAATG CACATGTTTG GCTCCTAGTG ATTTAATGGA CAGAGTTATC-3'
Y-chrom	5'-P-CAGGCCTGTA ATCCCAGCAA TAGTGATTTA CCTCAATGCA CATGTTTGGC TCCAAAAAAT ATGGATCTTG GCT-3'
ID 16	5'-TAMRA-CCTCAATGCT GCTGCTGTAC TAC-3'
ID 33	5'-FITC-CCTCAATGCA CATGTTTGGC TCC-3'

### Cells

Spreads of metaphase chromosomes were prepared from cultured lymphocytes obtained from peripheral blood from anonymous male donors. Culture of cells and preparation of chromosomes were performed according to standard techniques.

### Chromosome spreading

Metaphase chromosomes were spread in teflon-printed diagnostic well-slides according to the protocol presented in Figure 2.

### In situ detection

#### Restriction digestion

Restriction digestion was performed in 1× NEBuffer (NEB, Ipswich, MA, USA) (optimal for the enzyme), 0.2 μg/μl BSA (NEB) and 1u/μl restriction enzyme for 30 min at 37°C. NcoI (NEB) was used together with the Apo(a) probe and Dra I was used together with the Y-chrom probe. Slides were washed for 2 min at room temperature in 0.1 M Tris-HCL, 150 mM NaCl and 0.05% Tween-20 (wash buffer 1) and dehydrated through a series of ethanol (70%, 85% and 99%).

#### Lambda exonuclease

Exonuclease digestion was performed in a buffer containing 1× lambda exonuclease buffer (NEB), 0.2 μg/μl BSA (NEB) and 1u/μl lambda exonuclease (NEB) for 1 min at 37°C. Slides were washed for 2 min at room temperature in wash buffer 1 and dehydrated through a series of ethanol.

#### Hybridization and ligation

Hybridization and ligation were performed simultaneously in 1× T4 DNA ligase buffer (Fermentas, Vilnius, Lithuania), 0.5 μM probe and 0.1 u/μl T4 DNA ligase (Fermentas) for 30 min at 37°C. Slides were washed for 2 min at room temperature in wash buffer 1 followed by a wash in 2 × SCC and 0.05% Tween-20 for 5 min at 37°C and dehydrated through an ethanol series.

#### Rolling circle DNA synthesis

Rolling circle DNA synthesis was performed in a buffer containing 1× phi29 DNA polymerase buffer (Fermentas), 0.2 μg/μl BSA (NEB), 0.25 mM dNTPs and 1 u/μl phi29 DNA polymerase (Fermentas) for 30 min at 37°C. The slide was washed for 2 min at room temperature in wash buffer 1 and dehydrated through an ethanol series.

#### Detection

The rolling circle products were detected by hybridizing fluorescently labeled oligonucleotides in a buffer containing 20% formamide, 2× SSC, 5% glycerol and 0.17 μM of each of ID16 and ID33 for 30 min at 37°C. Slides were washed for 2 min at room temperature in wash buffer 1 and dehydrated through a series of ethanol and mounted with Vectashield (Vector Laboratories, Burlingame, CA, USA).

### Image analysis

Slides were analyzed with a Leica epifluorescence microscope (Leica, Wetzlar, Germany) and images were recorded with a SenSys CCD-camera operated by the SmartCapture 2 version 2.0 from Digitalscientific (Cambridge, UK). Leica 63× or 100× objectives were used for all images. Thresholding was performed using Adobe Photoshop (Adobe Systems).

## Competing interests

Target primed in situ detection was first presented in the patent application WO9720948. The granted patents are now part of the patent portfolio of the company In Situ RCP A/S in which the authors have financial interests.

## Authors' contributions

JSL designed the modified protocol described here, performed all experiments in the laboratory, and drafted the manuscript. MS participated in method development, probe design, figure design, data analysis and revision of the manuscript. JK initiated the project and contributed with analysis of the data and with critical revision of the manuscript. All authors read and approved the final manuscript.
